# Bilateral Diffuse Uveal Melanocytic Proliferation (BDUMP) associated with B-cell lymphoma: report of a rare case

**DOI:** 10.1186/s12885-015-1020-8

**Published:** 2015-01-30

**Authors:** Maria Pefkianaki, Rupesh Agrawal, Parul Desai, Carlos Pavesio, Mandeep S Sagoo

**Affiliations:** 1 Moorfields Eye Hospital, City Road, London, EC1V 2PD UK; 2 Biomedical Research Centre, Moorfields Eye Hospital NHS Foundation Trust and UCL Institute of Ophthalmology, London, UK; 3 National Healthcare Group Eye Institute, Tan Tock Seng Hospital, Singapore, Singapore; 4 St. Bartholomew’s Hospital, London, UK

**Keywords:** Bilateral diffuse uveal melanocytic proliferation, BDUMP, B-cell lymphoma

## Abstract

**Background:**

Bilateral diffuse uveal melanocytic proliferation (BDUMP) is a paraneoplastic ocular syndrome occurring in patients with systemic, often occult but advanced carcinoma and is the hallmark of poor prognosis. Ocular signs precede manifestation of systemic carcinoma by 3–12 months, highlighting the need for appropriate index of suspicion and prompt evaluation. Treatment options for BDUMP are limited. Investigations are aimed at finding the occult primary malignancy, which can be challenging. Modalities for treatment of the ocular findings include corticosteroids, surgery, external beam radiotherapy, and treatment of the underlying malignant neoplasm. However, it is uncertain whether earlier intervention for the systemic malignancy will impact survival, as this paraneoplastic phenomenon is thought to occur in advanced malignancy.

**Case presentation:**

We report a unique rare atypical case with BDUMP causing visual loss in a 62-year-old female as the presenting sign of central nervous system (CNS) B-cell lymphoma. Multiple grey or grey brown subretinal lesions with pigment clumps were present in both eyes on fundoscopy and multimodal imaging demonstrated multiple discrete lesions at the level of retinal pigment epithelium. Neuroimaging revealed presence of brainstem and cerebellopontine lesions suggestive of CNS lymphoma, which was further confirmed on biopsy.

**Conclusion:**

In the current atypical case, prompt diagnosis and immediate referral was key, with detailed systemic evaluation by an internist and oncologist. The reported case is distinct for the reason that BDUMP occurred secondary to primary CNS lymphoma, a hitherto unreported association.

## Background

Bilateral diffuse uveal melanocytic proliferation (BDUMP) is a paraneoplastic ocular syndrome occurring in patients with systemic, often occult but advanced carcinoma and is the hallmark of poor prognosis [1,2]. There are a total of less than 30 cases reported in world literature [3]. It is rarely reported in non-carcinomatous conditions, such as cervical leiomyoma [4].

The main characteristics are multiple round, red patches at the level of the retinal pigment epithelium (RPE), a striking pattern of multifocal areas of early hyperfluorescence corresponding with these patches, proliferation of choroidal nevus-like lesions as well as diffuse thickening of the uveal tract, exudative retinal detachment, and rapidly developing cataract [1]. In addition glaucoma, dilated episcleral vessels, iridocyclitis, shallow anterior chamber, ciliary body cysts, and iridodonesis have been reported [3].

Treatment options for BDUMP are limited. Investigations are aimed at finding the occult primary malignancy, which may be challenging. Treatment options for ocular involvement include corticosteroids, surgery, external beam radiotherapy, and management of the underlying malignant neoplasm [1,5].

Herein we report a unique rare case of BDUMP causing visual loss in an immunocompetent patient diagnosed with central nervous system (CNS) B-cell lymphoma -- a mode of atypical presentation that, to the best of our knowledge, has not been reported before.

## Case presentation

A 62-year-old female presented with a one-year history of visual disturbances and six-month history of reduction of vision in the left eye. Best corrected visual acuities were 6/6 in the right eye and 6/18 in the left eye with no signs of active or old inflammation in the anterior and posterior segments of both eyes. Mild posterior subcapsular cataract was noted in the left eye. Intraocular pressure was within normal limits.

**Figure 1:**
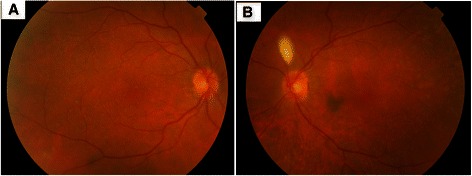
**Color fundus photograph showing presence of multiple round oval diffuse subretinal faint grey brown lesions in right (A) and left (B) eye.**

**Figure 2:**
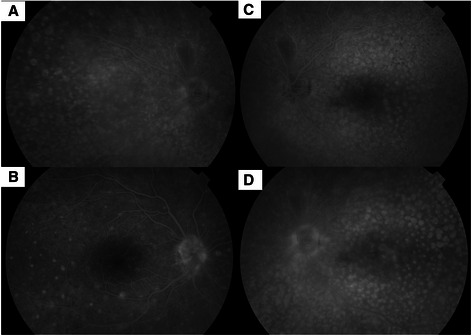
**Fundus fluorescein angiogram showing presence of hyperfluorescent lesions in right (A and B) and left (C and D) eye in early and late arteriovenous phase.**

**Figure 3:**
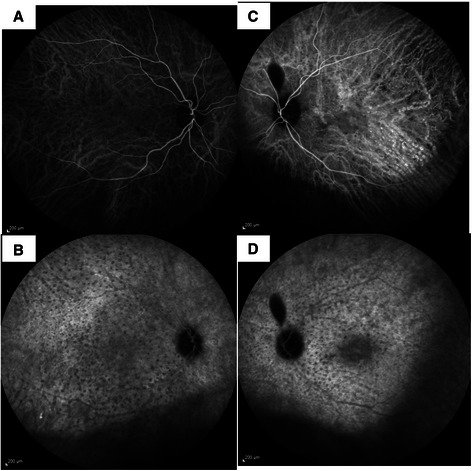
**Indocyanine green angiogram showing presence of numerous hypofluorescent lesions in right (A and B) and left (C and D) eyes.** The lesions remained hypofluorescent in very late frames as well **(B and D)**.

Gray or gray-brown lesions were noted at the level of the RPE with diffuse distribution in the fundus of both eyes (Figure 1). In addition, non-specific pigment clumps were present in the posterior pole of the left eye. Fluorescein angiography (FA) of both eyes showed the presence of multiple discrete and partially confluent hyperfluorescent lesions due to window defects (Figure 2). In the right eye these lesions were multiple hyperfluorescent areas involving the macula in both early (2A) and late (2B) arteriovenous phase. However, the lesions became more discrete and also reduced in number in late phase. A similar pattern was observed in the left eye (Figure 2C and 2D) but the number of lesions remained the same in the late phase. Indocyanine green angiography (ICG) demonstrated hypofluorescent spots from early to late phase, corresponding with multiple choroidal nevus-like lesions (Figure 3). There were fewer lesions in the right eye as compare to the left eye. Ultrasound B-scan did not show any elevated lesions.

**Figure 4:**
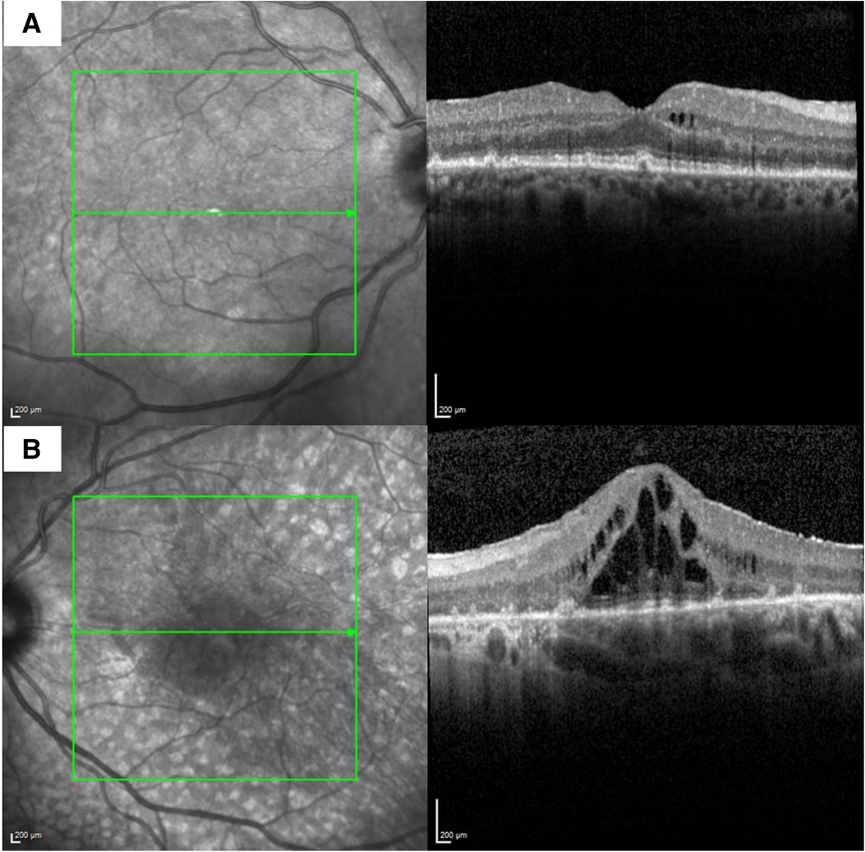
**Spectral domain OCT (Heidelberg) scans of right (A) and left (B) eyes showing presence of retinal pigment epithelial disturbances with excrescences at level of retinal pigment epithelium and presence of subretinal fluid in the left eye.**

**Figure 5:**
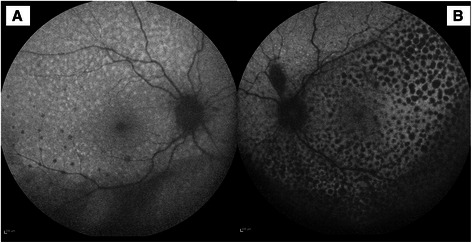
**Fundus autofluorescence of the right (A) and left (B) eye showing a greater number of hypofluorescent lesions as compared to the angiogram.**

Optical coherence tomography (OCT) demonstrated significant subfoveal fluid in the left eye as well as disruptions of the RPE in both eyes, corresponding to the plaque like areas on the fundus photographs and the areas of window defects on FA (Figure 4). Increased choroidal thickness (458 μm) was noted on OCT. Fundus autofluorescence showed complete absence of signals in these multiple nummular areas and also showed many more lesions as compare to angiographic images (Figure 5A and 5B). No electrodiagnostic studies were done, and also no serologic tests for paraneoplastic autoantibodies. HIV serology was negative.

The differential diagnosis included metastasis from occult primary or one of the forms of a white dot syndrome. The widespread lesions in both eyes were not typical of any of the inflammatory choriocapillaritis or stromal choroiditis disorders. Birdshot retinochoroidopathy was the closest of the inflammatory conditions but there was no retinal involvement to justify this entity and serum HLA29 was negative. Lesions were large for multiple evanescent white dot syndrome and too small for acute placoid multifocal pigment epitheliopathy. The differential diagnosis of bilateral primary intraocular lymphoma was also considered, though the characteristic subretinal and subretinal pigment epithelial deposits were absent.

Systemic evaluation revealed the presence of cerebellar symptoms. Neuroimaging showed brainstem and right cerebellopontine lesions suggestive of CNS lymphoma, which prompted MRI guided posterior fossa biopsy from the cerebellar peduncle. The biopsy showed large B cells that were CD20 positive, CD3 negative, CD79a positive, CD10 negative, bcl-6 positive, MUM1 positive, bcl-2 positive and EBER negative. Ki67 expression, which is an index of proliferation, showed 60% proliferation fraction in this area of biopsy. The histopathological findings confirmed diffuse large B-cell lymphoma of the brain with non-germinal centre subtype. The patient responded to a methotrexate-based systemic chemotherapy regimen, under the care of the oncologists. At 6 months, her ophthalmic condition remained stable on topical non-steroidal anti-inflammatory drops. Though the ocular lesion remains unresolved, the left macular oedema improved to give a final visual acuity of 6/12.

## Discussion

This unique case illustrates that BDUMP should be considered in the differential diagnosis of patients with atypical diffuse pigmented fundus lesions. The five cardinal signs of BDUMP described by Gass et al. [1] are: (1) multifocal, faintly visible, round or oval, red, subretinal patches, with (2) associated striking pattern of associated hyperfluorescence during the early phases of angiography; (3) development of multiple, slightly elevated, pigmented and non-pigmented uveal melanocytic tumors, as evidence of diffuse thickening of uveal tract; (4) exudative retinal detachment; and (5) rapid progression of cataract [1].

Our case demonstrated some of these features, indicating an earlier stage of BDUMP, before the onset of exudative retinal detachment and progression of cataract. However, multifocal, faintly visible, roundish oval subretinal lesions, which were hyperfluorescent on FA suggested the diagnosis of BDUMP. The precise correspondence of the focal areas of early hyperfluorescence and the subretinal patches suggest that they are fenestrations of the pigment epithelium caused by its depigmentation and focal destruction in the absence of RPE and excrescences in inner segment-outer segment (IS-OS) junction demonstrated the level of lesions in the RPE. The same was also demonstrated in our case on spectral-domain OCT scan. To our knowledge, this case is distinct in the literature in demonstrating those changes in RPE on real time non-invasive scans. The other possible differential diagnoses of choriocapillaritis or stromal choroiditis were less likely as there were no classical clinical signs of any of those entities. Birdshot retinochoroidopathy was the closest differential diagnosis but due to lack of classical clinical and angiographic signs, the diagnosis of masquerade syndrome was kept and hence the patient was investigated for possible systemic association. Lymphoma-associated retinopathy as described by To et al. [6], was a pigmentary retinopathy occurring on the onset of chemotherapy for Hodgkin lymphoma. Our case had pale gray brown nummular lesions, rather than retinitis pigmentosa-like pigmentary migration of lymphoma-associated retinopathy. Our case fits in with BDUMP features as described by Gass: multifocal, faintly visible, round or oval, red, subretinal patches, with associated striking pattern of associated hyperfluorescence during the early phases of angiography [1].

Two types of melanocytes are found in the normal uveal tract: the normal melanocytes and, in many patients, nevus cells. Normal melanocytes are nonreactive cells that rarely, if ever, proliferate. Nevus cells that are present, either focally or diffusely (melanocytosis), do have the capability of proliferation that, in part, is subject to hormonal control and that normally is limited to the prepubertal and early adult years [7].

Suggestions for the pathogenesis of BDUMP have been postulated as either the *de novo* development of uveal melanocytic proliferation and the systemic carcinoma in response to a common oncogenic stimulus, [8] or the *de novo* development of uveal melanocytic proliferation in response to a hormone-secreting visceral carcinoma, or possibly coincidental development of bilateral low-grade diffuse uveal melanomas and a systemic carcinoma in patients genetically predisposed to neoplasia [1]. In addition, Miles et al. have recently shown that there is cultured melanocytic elongation and proliferation factor (CMEP) factor, in the serum that causes uveal melanocytes to proliferate [7].

Systemic carcinomas reported with BDUMP are ovarian, lung, gall bladder, cervical, uterine, kidney, pancreatic, breast, esophageal, and colorectal cancers. They may also be associated with melanocytic proliferation in other tissues [8,9]. Although rare, BDUMP has consistently resulted in devastating visual consequences. Usually during the year preceding death, patients with this paraneoplastic syndrome have severe bilateral vision loss. Vision decline has been attributed to destruction of photoreceptors and underlying RPE, serous retinal detachments, and, later, cataracts [1].

In the current case, the prompt diagnosis and immediate referral was key, with detailed systemic evaluation by internists and oncologists. As per the original description by Gass et al. [1] ocular signs precede manifestation of systemic carcinoma by 3–12 months, highlighting the need for the appropriate index of suspicion and prompt evaluation [10]. It is uncertain whether earlier intervention for the systemic malignancy will impact survival, as this paraneoplastic phenomenon is thought to occur in advanced malignancy.

Treatment of BDUMP is usually directed towards the primary cancer. Although transient visual improvement has been reported in patients with BDUMP in response to systemic corticosteroids and chemotherapy, [9] most cases have shown no effect of corticosteroid therapy alone [3] and no significant effect of radiotherapy [1]. Our case responded to systemic chemotherapy along with topical non-steroidal anti-inflammatory drops.

## Conclusions

This atypical case of BDUMP is unique for several reasons. Although it had the presence of subretinal lesions, which were hyperfluorescent on FA, exudative retinal detachment or cataract were absent, indicating perhaps an earlier stage in the evolution of this condition. Furthermore, we documented OCT changes similar to reported histopathological changes in BDUMP [10]. Finally, this case is distinct for the reason that BDUMP occurred secondary to primary CNS lymphoma, a hitherto unreported association.

### Consent

Written informed consent was obtained from the patient for publication of this Case report and any accompanying images. A copy of the written consent is available for review by the Editor-in-Chief of this journal. This report adhered to the tenets of the Declaration of Helsinki.

## Abbreviations

BDUMP: Bilateral diffuse uveal melanocytic proliferation
CNS: Central nervous system
RPE: Retinal pigment epithelium
FA: Fluorescein angiography
OCT: Optical coherence tomography
MRI: Magnetic resonance imaging
IS-OS: Inner segment-o uter segment
ICG: Indocyanine green angiography
CMEP: Cultured melanocytic elongation and proliferation factor
